# Clinical Experience With a Dedicated Neurocritical Care Quality Improvement Program in an Academic Medical Center

**DOI:** 10.7759/cureus.52730

**Published:** 2024-01-22

**Authors:** Abhijit V Lele, Annika S Bhananker, Christine T Fong, Christine Imholt, Andrew Walters, Ellen F Robinson, Michael J Souter

**Affiliations:** 1 Neurocritical Care/Anesthesiology, Harborview Medical Center, Seattle, USA; 2 Anthropology, Rice University, Houston, USA; 3 Anesthesiology and Pain Medicine, University of Washington, Harborview Medical Center, Seattle, USA; 4 Anesthesiology and Pain Medicine, Harborview Medical Center, Seattle, USA; 5 Quality Improvement, Harborview Medical Center, Seattle, USA

**Keywords:** trauma, performance measures, quality metrics, outcomes, process, healthcare outcomes, process controls, structure, neurocritical care unit, quality improvement (qi)

## Abstract

Background

Managing neurocritical care patients encompasses many complex challenges, necessitating specialized care and continuous quality improvement efforts. In recent years, the focus on enhancing patient outcomes in neurocritical care may have led to the development of dedicated quality improvement programs. These programs are designed to systematically evaluate and refine care practices, aligning them with the latest clinical guidelines and research findings.

Objective

To describe the structure, processes, and outcomes of a dedicated Neurocritical Care Quality Improvement Program (NCC-QIP) at Harborview Medical Center, United States; a quaternary academic medical center, level I trauma, and a comprehensive stroke center.

Materials and methods

We describe the development of the NCC-QIP, its structure, function, challenges, and evolution. We examine our performance with several NCC-QI quality measures as proposed by the Joint Commission, the American Association of Neurology, and the Neurocritical Care Society, self-reported quality improvement (QI) concerns and QI initiatives undertaken because of the information obtained during our event/measure reporting process for patients admitted between 1/1/2014 and 06/30/2023.

Results

The NCC-QI reviewed data from 20,218 patients; mean age 57.9 (standard deviation 18.1) years, 56% (n=11,326) males, with acute ischemic stroke (AIS; 22.3%, n=4506), spontaneous intracerebral hemorrhage (ICH; 14.8%, n=2,996), spontaneous subarachnoid hemorrhage (SAH; 8.9%, n=1804), and traumatic brain injury (TBI; 16.6%, n=3352) among other admissions, 37.4% (n=7,559) were mechanically ventilated, and 13.6% (n=2,753) received an intracranial pressure monitor. The median intensive care unit length of stay was two days (Quartile 1-Quartile 3: 2-5 days), and the median hospital length of stay was seven days (Quartile 1-Quartile 3: 3-14 days); 53.9% (n=10,907) were discharged home while 11.4% (2,309) died. The three most commonly reported QI concerns were related to care coordination/communication/handoff (40.4%, n=283), medication-related concerns (14.9%, n=104), and equipment/devices-related concerns (11.7%, n=82). Hospital-acquired infections were in the form of ventilator-associated pneumonia (16.3%, n=419/2562), ventriculostomy catheter-associated infections (4%, n=102/2246), and deep venous thrombosis/pulmonary embolism (3.2%, n=647). The quality metrics documentation was as follows: nimodipine after SAH (99.8%, 1802/1810), Hunt and Hess score (36%, n=650/1804), and ICH score (58.4% n=1752/2996). In comparison, 72% (n=3244/4506) of patients with AIS had a documented National Institute of Health Stroke Scale. Admission Glasgow Coma Score was recorded in 99% of patients with SAH, ICH, and TBI. Educational modules were implemented in response to event reporting.

Conclusion

A dedicated NCC-QIP can be successfully implemented at a quaternary medical medical center. It is possible to monitor and review a large volume of neurocritical care patients, The three most reported NCC-QI concerns may be related to care coordination-communication/handoff, medication-related concerns, and equipment/devices-related complications. The documentation of illness severity scores and stroke measures depends upon the type of measure and ability to reliably and accurately abstract and can be challenging. The quality improvement process can be enhanced by educational modules that reinforce quality and safety.

## Introduction

Neurocritical care (NCC) is a specialized critical care discipline that focuses on managing patients with acute brain injury, including traumatic brain injury and stroke, among others [1,2]. Continuous quality improvement (CQI) is integral to neurocritical care delivery. It is a core element of a level I neurocritical care unit (the highest designation among all neurocritical care units) [3]. A survey of neurocritical care providers found that less than 50% of neurocritical care programs report the presence of a dedicated neurocritical care quality improvement (NCC-QI) program [4].

Establishing a dedicated NCC-QIP has become increasingly crucial for enhancing patient outcomes, ensuring adherence to best practices, and addressing the complex challenges inherent in this field. This study examines the NCC-QIP at Harborview Medical Center, a quaternary academic medical center. The NCC-QIP was developed to optimize patient care and outcomes in neurocritical care settings. The program is structured to systematically review and improve various aspects of care, including patient management, treatment protocols, and interdisciplinary communication. It focuses on analyzing a range of quality improvement (QI) measures proposed by prominent organizations such as the Joint Commission [5-7], the American Association of Neurology [8], and the Neurocritical Care Society [9].

Describing the structure, processes, and outcomes of a dedicated NCC-QIP is crucial for several reasons. First, it provides a clear framework for best practices in neurocritical care, illustrating effective organizational and resource allocation strategies. This description enables the evaluation and continuous refinement of care processes, identifying areas for improvement and ensuring the delivery of high-quality care. Additionally, by documenting outcomes, the program can objectively measure its effectiveness in enhancing patient care, such as improving clinical documentation and survival rates, reducing complications, and facilitating recovery. Such a comprehensive review is a valuable model for other institutions looking to establish or enhance their neurocritical care services, offering insights into potential challenges and effective strategies. It also ensures consistency and standardization in care across different providers, a critical factor in reducing variability and enhancing treatment quality. Furthermore, this detailed description contributes to research and development in the field, informing future studies and innovations. It aids in policymaking and meeting accreditation standards while engaging various stakeholders by providing a transparent overview of the program's operations and achievements. In essence, the thorough description of an NCC-QIP's structure, processes, and outcomes is vital to advancing neurocritical care and ensuring superior patient care.

Hence, this study aimed to 1) describe the development, structure, function, and evolution of the NCC-QIP, 2) describe the performance of the NCC-QIP against several neurocritical care quality improvement measures, 3) examine the self-reported QI concerns, and 4) describe the initiatives undertaken in response to data obtained from event and measure reporting processes.

## Materials and methods

Institutional review board

The study was conducted in accordance with the Declaration of Helsinki and approved by the Institutional Review Board of the University of Washington (STUDY00009382, approved on 7/2/2023). A waiver of consent was provided.

Study design and clinical setting 

This was a single-center retrospective review of the structure, process, and outcomes of patients admitted to the neurocritical care service (NCCS) at Harborview Medical Center, a level I trauma and comprehensive stroke center. The multidisciplinary NCCS comprises board-certified/eligible neurointensivists from the departments of Anesthesiology and Pain Medicine, Neurology, Neurological Surgery, Emergency Medicine, and the division of Pulmonary Critical Care Medicine. The NCCS team is comprised of neurointensivists, advanced practice providers, and trainees from anesthesiology, neurology, neurological surgery, emergency medicine, pulmonary critical care, anesthesiology critical care, and neurocritical care fellows, as well as medical students who rotate for a clerkship in the neurocritical care unit.

Study period 

The study reviewed the inception of the NCC-QIP and key milestones between 2008 and 2023. In addition, the study examines admissions to the neurocritical care service between 1/1/2014 and 06/30/2023, during which time a dedicated neurocritical care outcomes database was formed.

Inclusion and exclusion

Patients 18 years and older admitted to the neurocritical care service during the study period were included.

Data collection

We queried the electric medical records of all patients admitted to NCCS between 1/1/2014 and 06/30/2023. Demographical data included age, sex, race/ethnicity, insurance carrier, admitting diagnosis, admitting Glasgow Coma Scale score, mechanical ventilation, and intracranial pressure monitoring [10]. Admitting diagnosis was categorized into the following: acute ischemic stroke, brain tumors, meningitis, neuromuscular disease, post-op after procedures on the spine, post-op ICU care after intracranial procedures, spontaneous intracerebral hemorrhage, status epilepticus/seizures, spontaneous subarachnoid hemorrhage, traumatic brain injury, traumatic spinal cord injury, and cerebrospinal fluid shunt malfunction/infection/hydrocephalus.

Outcomes

The primary outcomes were performance with the neurocritical care quality measures proposed by the Joint Commission [5-7], the Neurocritical Care Society [9], and the American Academy of Neurology [8]. Since each measure includes a numerator and a denominator and a combination of automated (and sometimes manual) data abstraction, we chose to report selected quality measures for whom we have been able to validate data. Eleven neurocritical care quality metrics were analyzed for trends between 2014 and 2023. Yearly data were presented as line graphs with 80% benchmarking for the metrics with data documentation and 5% for hospital-acquired infections/Agency for Healthcare Research and Quality/Patient-Safety Indicators (AHRQ-PSI) [11,12] deep venous thrombosis/pulmonary embolism. We also highlight that in the year 2021, the electronic health records were migrated to a new vendor. We think this is important to note since electronic health record platforms may change how data is entered and abstracted and could influence the documentation rates of specific quality indicators. The quality metrics/performance measures studied are as follows:

1) Documentation of the administration of nimodipine for aneurysmal subarachnoid hemorrhage (numerator = patients with documentation of the administration of nimodipine within the first 24 hours of diagnosis of aneurysmal subarachnoid hemorrhage, denominator = all patients with aneurysmal subarachnoid hemorrhage) [13].

2) Documentation of the Hunt and Hess score in patients with aneurysmal subarachnoid hemorrhage (numerator = patients with documentation of Hunt and Hess within six hours of admission to the emergency room and before surgical intervention, denominator = all patients with aneurysmal subarachnoid hemorrhage [14].

3) Documentation of intracerebral hemorrhage (ICH) score in patients with spontaneous ICH (numerator = patients with documentation of ICH score within six hours of admission to the emergency room and before surgical intervention, denominator = all patients with spontaneous intracerebral hemorrhage) [14].

4) Documentation of acute ischemic stroke severity score (National Institute of Health Stroke Scale, NIHSS), (numerator patients with a documented NIHSS, denominator = patients admitted with acute ischemic stroke) [15].

5) Documentation of admission Glasgow Coma Score in patients with SAH, ICH, and TBI [10].

6) Hospital-acquired infections include catheter-associated urinary tract infections [16], ventilator-associated pneumonia [17], and ventriculostomy-associated infections [18].

8) Agency for Healthcare Research and Quality-Patient Safety Indicators [18] such as deep venous thrombosis/pulmonary embolism.

The diagnoses were confirmed by codification using either the International Classification of Diseases (ICD) version 9: ICD-9 [19] for patients admitted between 2014 and 2015 and version 10:ICD-10 [20] for patients admitted between 2016 and 2023.

Data analysis

Descriptive analysis described the study sample. Categorical data were reported as counts and percentages. After performing the normality testing using the Shapiro-Wilk test [21], the continuous data were reported as mean (standard deviation) or median (interquartile range, Quartile 1-Quartile 3). RStudio was used for statistical analysis [22]. STATA version 15 and Tableau were used for the creation of graphs [23].

**Figure 1:**
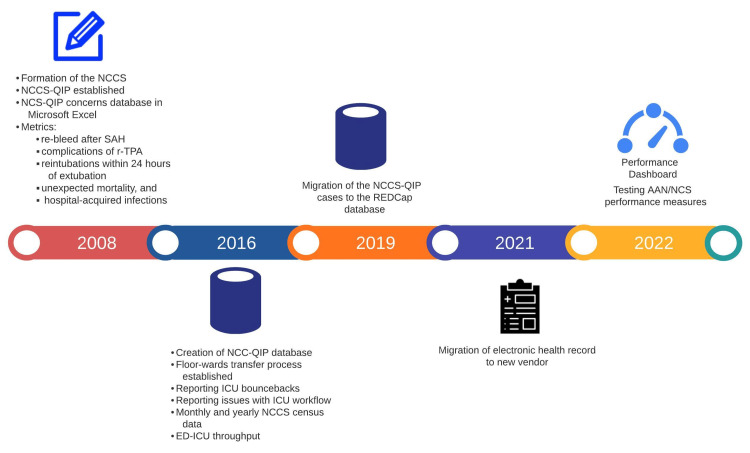
The evolution of Neurocritical Care/Critical care quality measures tracked and reported at Harborview Medical Center NCCS: neurocritical care service; NCC-QIP: neurocritical care quality improvement program; SAH: spontaneous subarachnoid hemorrhage; r-TPA: recombinant tissue plasminogen activator; AAN: American Academy of Neurology; NCS: Neurocritical Care Society: ED: emergency department; REDCap: Research Electronic Data Capture

Figure 1 highlights the evolution of the NCC-QIP quality measures tracked and reported at Harborview Medical Center.

The process of the intake, processing, reporting, and dissemination of the review of self-reported NCC-QI concerns

Since its launch, a concerted effort has been put together to maintain a confidential quality database to track, monitor, report, and disseminate NCC-QI concerns. In its first iteration, this was done as self-reported QI cards filled out by NCCS providers reported quality concerns about any aspect of NCC delivery. This innovative approach allowed for identifying potential areas of improvement and fostering a culture of continuous growth and development within the NCC program. This approach also allowed for reviews in close to real-time when the teaching is more meaningful than retrospective learning.

As the program progressed, it became evident that a more systematic and structured approach to QI was necessary to ensure the ongoing success of the NCC-QIP. This realization led to the integration of an electronic database entry system [24]. This strengthened the maintenance of confidentiality, along with an easy reporting mechanism.

**Figure 2:**
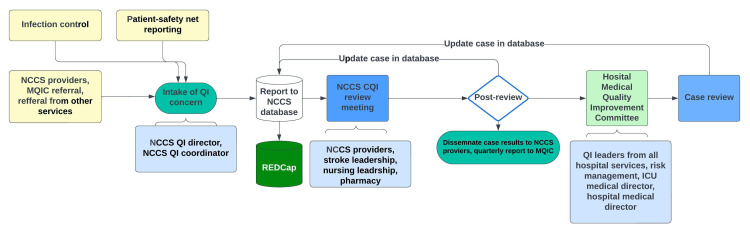
A process map of intake, processing, reporting, and dissemination of neurocritical care quality concerns. NCCS: neurocritical care service; QI: quality improvement; ICU: intensive care unit

Figure 2 is a process map of the intake, processing, reporting, and dissemination of NCC-QI concerns.

Recurring meetings dedicated to NCC-QI

Discussion amongst NCCS stakeholders is an essential component of maintaining the integrity of the NCC-QI program. This is accomplished via recurring meetings (hybrid, in-person, teleconferencing), which invite all members of NCCS along with stakeholders from intensive care nursing leadership, stroke QI leadership, and neurological surgical QI leadership, along with pharmacists. Trainees are encouraged to attend this meeting. During the meeting, the NCC-QI director presents the NCC-QI quality dashboard. This is followed by case discussions, for which the providers intimately familiar with the case are invited, and a non-punitive, non-blame approach is used to identify systems-level opportunities for growth. Each case is assigned a score. Following the review, the NCC-QI director collates all NCC-QI cases to be reported to the hospital's medical quality committee (MQIC) every quarterly. In parallel, the NCC-QI director will also independently review unexpected mortality cases and cases requested by the hospital's medical quality committee (MQIC) or other hospital services. The NCC-QI director attends monthly MQIC meetings to provide input on cases related to NCCS.

## Results

Sample characteristics

Table 1 summarizes the patients admitted to the neurocritical care service between 1/1/2014 and 06/30/2023. The NCC-QIP reviewed data from 20,218 patients: mean age 57.9 (SD 18.1) years, 56% (n=11,326) males, with acute ischemic stroke (AIS; 22.3%, n=4506), spontaneous intracerebral hemorrhage (s-ICH; 14.8%, n=2,996), spontaneous subarachnoid hemorrhage (SAH; 8.9%, n=1804), and traumatic brain injury (TBI; 16.6%, n=3352) among other admissions. Thirty-seven point four percent (37.4%; n=7,559) were mechanically ventilated, and 13.6% (n=2,753) received an intracranial pressure monitor. The median intensive care unit length of stay was two days (Q1-Q3 2-5 days); 53.9% (n=10,907) were discharged home while 11.4% (2,309) died.

**Table 1:** Characteristics of patients admitted to the neurocritical care service between 1/1/2014 and 06/30/2023 SD: standard deviation; ICU; intensive care unit

Patient characteristics	Overall (N=20,218)
Age in years Mean (SD)	57.9 (SD 18.1)
Sex	
Female	8892 (44.0%)
Male	11326 (56.0%)
Admitting diagnosis	
Acute ischemic stroke	4506 (22.3%)
Brain tumors	314 (1.6%)
Meningitis	26 (0.1%)
Neuromuscular disease	9 (0.0%)
Other	1320 (6.5%)
Post-op after spinal procedures	1110 (5.5%)
Post-op ICU care for cranial procedures	3209 (15.9%)
Spontaneous intracerebral hemorrhage	2996 (14.8%)
Status epilepticus/seizures	533 (2.6%)
Subarachnoid hemorrhage	1804 (8.9%)
Traumatic brain injury	3352 (16.6%)
Traumatic spinal cord injury	918 (4.5%)
Cerebrospinal fluid shunt malfunction/infection/Hydrocephalus	121 (0.6%)
Race/ethnicity	
Non-White	4410 (21.8%)
White	15794 (78.1%)
Missing	14 (0.1%)
Preferred language	
English	18166 (89.9%)
Non-English	2032 (10.1%)
Missing	20 (0.1%)
Mechanical ventilation	7559 (37.4%)
Intracranial pressure monitoring	2753 (13.6%)

**Figure 3:**
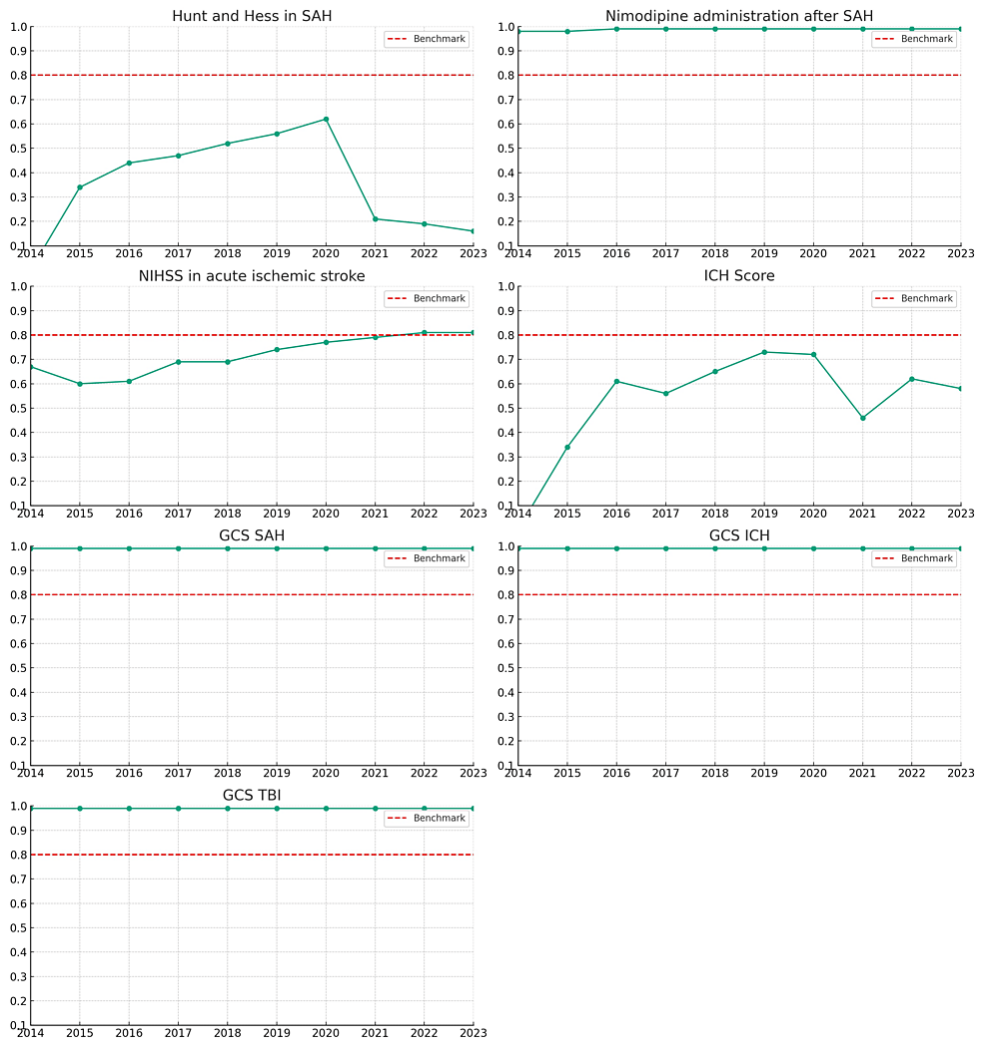
Critical care/NCC-QI quality metrics/performance measures dashboard NCC-QI: Neurocritical Care Quality Improvement Program; SAH: spontaneous subarachnoid hemorrhage; ICH: spontaneous intracerebral hemorrhage; AIS: acute ischemic stroke; TBI: traumatic brain injury; GCS: Admission Glasgow Coma Scale score The Y-axis should be read as between 0% and 100%, with the metric benchmark set at 80%.

Figure 3 displays the trends in the NCC-QIP quality measures reviewed during the study period. SAH measures were as follows: documentation of nimodipine (99.8%, 1802/1810) and the Hunt and Hess score (36%, n=650/1804). ICH score was documented in 58.4% (1752/2996). In comparison, 72% (n=3244/4506) of patients with AIS had a documented National Institute of Health Stroke Scale. Admission Glasgow Coma Score was recorded in 99% of patients with SAH, ICH, and TBI. Of the seven metrics displayed in Figure 3, consistently above benchmark documentation, was found for nimodipine administration in the first 24 hours in SAH and admission Glasgow Coma Scale score in patients with AIS, IPH, and SAH. The documentation of NIHSS for AIS showed a steady uptrend in meeting the benchmark while the documentation of the Hunt and Hess and ICH scores was below the benchmark.

**Figure 4:**
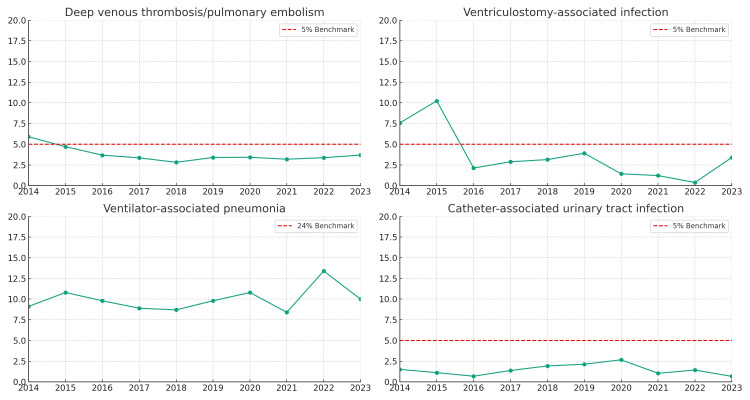
Neurocritical care outcomes dashboard The Y-axis should reach 0-20%. The benchmark was set at 5% except for ventilator-associated pneumonia, which should read 24%.

Figure 4 displays the trends in deep venous thrombosis/pulmonary embolism, ventriculostomy-associated infections, ventilator-associated pneumonia, and catheter-associated urinary tract infections during the study period. Hospital-acquired infections were in the form of ventilator-associated pneumonia (16.3%, n=419/2562), ventriculostomy catheter-associated infections (4%, n=102/2246), and deep venous thrombosis/pulmonary embolism (3.2%, n=647). We observed low rates of deep venous thrombosis/pulmonary embolism, ventriculostomy-associated infections, and catheter-associated urinary tract infections but consistently high ventilator-associated pneumonia rates.

**Figure 5:**
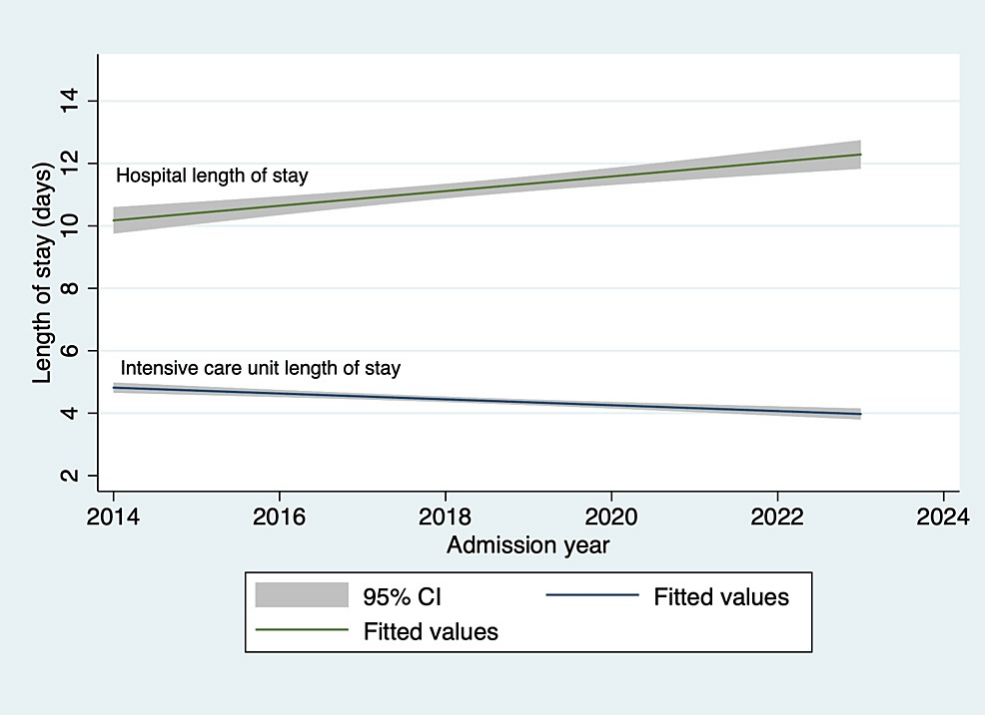
Intensive care unit and hospital length of stay in days over the study period CI: 95% confidence intervals, data adjusted for admission Glasgow Coma Scale score

Figure 5demonstrates the intensive care unit and hospital length of stay trends. Notably, the coefficient of determination = 0.869, and the p-value is <0.001, indicating a very strong linear relationship between the admission year and the intensive care unit length of stay. Similarly, the hospital length of stay with a coefficient of determination of 0.450 and a p-value of 0.0337, indicative of a moderate linear relationship.

**Figure 6:**
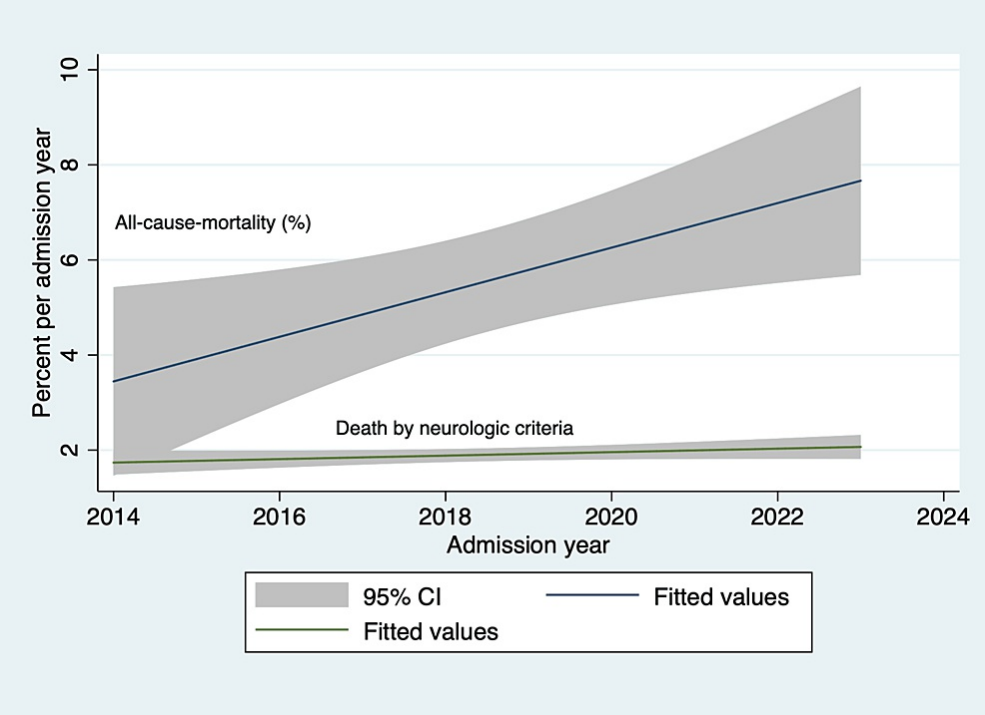
Mortality trends over the study period adjusted by admission Glasgow Coma Scale score CI: 95% confidence interval

Figure 6 highlights trends in risk-adjusted (admission Glasgow Coma Scale score) mortality over the study period. The coefficient of determination is 0.515, and the p-value is 0.0195, indicating a moderate linear relationship between the year and all-cause mortality. The trends in mortality by declaration of death by neurologic criteria have remained fairly stable.

Self-reported neurocritical care QI concerns 

**Figure 7:**
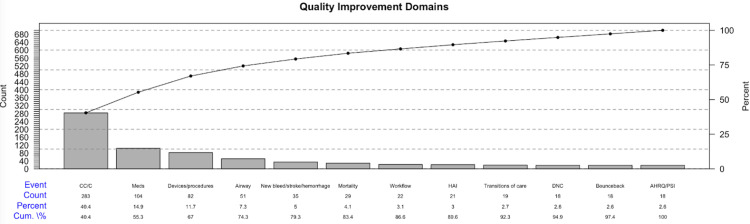
Pareto diagram with self-reported CC/NCC-QI concerns NCC-QIP: neurocritical care quality improvement program; CC/C: care-coordination/communication, Meds: medication-related concerns; HAI: hospital-acquired infections; DNC: death by neurologic criteria; AHRQ/PSI: Agency for Healthcare Research and Quality: patient safety indicators

Figure 7 is a Pareto diagram of the 809 self-reported NCC-QI concerns reported to the NCCS QI database. Of note, the three most commonly reported QI concerns were related to care coordination-communication/handoff (40.4%, n=283), medication-related concerns (14.9%, n=104), and equipment/devices-related concerns (11.7%, n=82).

Quality-improvement initiatives undertaken during the study period 

Table 2 highlights QI initiatives undertaken by NCCS. These initiatives were undertaken as a result of self-reported QI events that prompted a root-cause analysis, leading to systems-level changes.

**Table 2:** Quality improvement initiatives undertaken by the Neurocritical Care Service at Harborview Medical Center NCCS: neurocritical care service; CAUTI: catheter-associated urinary tract infection; ICU: intensive care unit

Domain	Initiatives
Care-coordination communication	Paging communication between nurses and NCCS providers, communication checklist during the transition to comfort measures only, integration of cultural mediator for NCCS patients. Development of an educational module to educate rotating trainees about the importance of integrating cultural mediators for NCCS patients. Reinforcing standards of intra-team and inter-team communication.
Medication-associated events	Reinforcing medication safety related to safe interruption and resumption of chemical venous thromboembolism prophylaxis in patients undergoing neurosurgical and non-neurosurgical procedures, the inclusion of pharmacists in daily rounds.
Device-associated complications	Implementation of an indication-based cerebrospinal fluid sampling in patients with spontaneous SAH who have an indwelling external ventricular drain; fostering clinical proficiency in the quality and safety related to cerebrospinal fluid diversion devices through an EVD educational module. Changing policy around monitoring intracranial pressure monitoring during intrahospital transport of critically ill patients.
Death by neurologic criteria	Development of an educational module regarding death by neurologic criteria, complemented by simulation sessions and bedside/classroom teaching.
Mortality	Prompt mortality review in a multidisciplinary setting.
Hospital-acquired infections	CAUTI initiative for early Foley catheter removal and Foley catheter reinsertion algorithm.
Transitions of care	Structured in-person handoff for transfer of care between the ICU and the floor/ward team.
Stroke performance measures	Revising electronic data abstraction for stroke severity measures by creating a smart phrase in ICU progress notes as well as creating data columns on electronic medical record patient lists for frontline ICU providers to review any missing data Recurring reminders to frontline providers to document illness severity scores.

## Discussion

This study explored the structure, process, and outcome of a dedicated neurocritical care quality improvement program at Harborview Medical Center. The main findings of the study are: 1) The NCC-QIP successfully monitored and reviewed a significant patient population, demonstrating its capacity to handle high volumes of complex cases; 2) The three most commonly reported QI concerns were related to care coordination/communication/handoff, medication-related concerns, and equipment/devices-related complications; 3) The documentation of nimodipine in the first 24 hours of admission after SAH was high, but the documentation of Hunt and Hess score, ICH score was low; 4) The documentation of admission Glasgow Coma Scale score was high, 5) The documentation of NIHSS was improving in patients with acute ischemic stroke, and 6) Quality improvement initiatives including educational modules were launched in response to the event reporting process and included reinforcement on and revision to existing protocols.

Monitoring a very high volume of patients

On average, our neurocritical care service admits more than 1800 patients yearly. When the NCC-QIP database was being developed, we started with one year of patients and validated information such as patient demographics, admitting diagnosis, ICU and hospital length of stay, and discharge disposition. Over the study period, the database has grown from 1800 to more than 20,000 patients. The number of variables that can be studied has also increased to upwards of 150. The information technologist expert analyst, author CTF, and author AVL regularly check in to identify issues with existing and new data variables. A random set of patient-level data is manually reviewed to confirm. If there are other data sources, such as the operating room encounters, data merge is used to validate data points in the NCCS database. We have harnessed the power of Structured Query Language (SQL) and RStudio [22] to provide summary statistics and report queries. Such a process has enabled us to continue monitoring a very high volume of patients admitted to the neurocritical care service.

Self-reported quality concerns 

We observed that care coordination/communication/handoff, medication-related concerns, and equipment/devices-related complications were the most cited QI domains. The results of our study are similar to a recently published study in the field of neuroanesthesiology and perioperative neurosciences [25]. One of the first QI initiatives undertaken by our service was that of communication between the bedside nurses and the members of the neurocritical care service. Through a survey and a team-building session, we were able to arrive at a consensus-based approach to the safe, efficient, and timely escalation of patient-level concerns to healthcare providers [26]. The self-reporting of these concerns also highlights the strong commitment from healthcare providers to improve the process of care coordination and communication. Medication-related concerns are equally crucial in neurocritical care as in other healthcare areas. Creating an onboarding process and reinforcing our policies and procedures around safe initiation and interruptions in chemical venous thromboembolism prophylaxis was a significant focus of the neurocritical care service. This included the creation of a hospital-wide clinical decision-making tree outlining the consensus-based approach to venous thromboembolism prophylaxis and treatment. External ventricular drain-associated QI concerns were primarily related to a lack of routine intracranial pressure monitoring during intrahospital transport, which seems to be a prevalent problem worldwide [27]. As a result of patient safety events as well as research [28] and to adhere to the recommendations from the Society for Neuroscience in Anesthesiology and Critical Care [29], there has been a change in hospital policy late in 2023, whereby routine monitoring of intracranial pressure during intrahospital transport is now a standard of care at Harborview Medical Center. Similarly, in patients with aneurysmal SAH, we have moved away from routine twice-weekly cerebrospinal fluid sampling to indication-based sampling based on the recommendations of the Infectious Disease Society for Healthcare-associated Ventriculitis and Meningitis America [30]. Since the change, we have noticed an 88% reduction in routine cerebrospinal fluid sampling yet maintaining our low rate of ventriculitis.

Documentation of illness severity scores and trends in length of stay, mortality, and complications 

The illness severity scores are reported to the Joint Commission [7]. Face validity for the documentation is good; however, the Hunt and Hess and ICH scores must be demonstrated within six hours of arrival to the emergency department and before any surgical intervention such as placement of EVD. This is where we continue to struggle. As is evident from our trend analysis, we have yet to go beyond the 80% benchmark, although we are close to it. Introducing a new electronic medical record system proved to be a significant challenge to overcome. The author, AVL, the stroke coordinator, and the stroke leadership team put together a new initiative in late fall of 2023, one where we created a smart phrase that automatically pulls illness severity scores into a daily progress note. This accomplishes two primary goals. An empty report on the progress note indicates that the stroke navigator where these measures reside is incompletely filled out. We also added the stroke illness severity column to the list of visible variables that clinicians can see when they open the list of patients on the neurocritical care service. While early reports of this implementation are encouraging, we will continue to monitor the sustainability of accurately documenting these scores in compliance with the Joint Commission's recommendations. The trends in the overall intensive care unit length of stay are encouraging, with a linear reduction in the length of stay similar to prior published literature [31]. However, the trends for hospital length of stay and adjusted mortality, especially after the advent of the coronavirus disease 2019 (COVID-19) pandemic, need further examination (comparison by admission diagnosis, insurance carrier, economic and social factors, and insurance-specific policies) to find opportunities for quality improvement. Similarly, the trends in the rates of catheter-associated urinary tract infections and deep venous thrombosis/pulmonary embolism are encouraging. The ventilator-associated pneumonia rates are high but below nationally reported numbers [17].

Quality improvement initiatives 

Table 2 shows several quality improvement initiatives implemented after root cause analysis of several patient safety events. Specifically, the integration of the caseworker cultural mediator was a QI initiative that was initially successful [32]. The purpose was to align care to the religious, social, and cultural beliefs and values of patients with non-English-language preferences. Due to the inability to sustain the progress, we added the caseworker cultural mediator educational module [33], which complements the already established workflow by reminding the trainees and advanced practice providers about the importance of integrating cultural mediators in the care of these families. Trainees, attending physicians, advanced practice providers, bedside nurses, and respiratory therapists have unique roles in declaring death by neurologic criteria (DNC). The DNC educational module [34] was set up to educate our frontline providers regarding the basics of assessment for brain death and navigate common and complex nuances related to pre-requisites, clinical examination, apnea trial, and ancillary testing. Similarly, a module was created to educate clinicians about quality and safety practices for managing patients with external ventricular drains [35]. These educational modules are mandatory for all providers of neurocritical care services and form part of their onboarding process. In addition to these online modules, we have incorporated simulation sessions for EVD training and DNC, with classroom and bedside teaching to review the core concepts.

Challenges faced by the Neurocritical Care Quality Program

Implementing a quality improvement program in a complex medical environment is challenging. Limited resources and budgetary restrictions can hinder the development and implementation of quality improvement initiatives. The NCC-QIP has had to prioritize projects and interventions, focusing on those with the highest potential for impact on patient outcomes and safety. Change can be resisted by healthcare professionals who may be accustomed to established practices and protocols. Stakeholder buy-in and creating a shared mental model with interactive changes have been critical to our program's success. The NCC-QIP has had to work diligently to foster a culture of continuous improvement and demonstrate the benefits of adopting new approaches and technologies. Ongoing education and training are essential to ensure the success of quality improvement initiatives. The NCC Quality Program must continue to allocate resources to provide training sessions, workshops, and educational materials to support adopting new practices and maintain staff competency. Keeping the engagement and involvement of trainees and faculty can be challenging in a busy clinical environment. The NCC-QIP has focused on developing strategies to encourage participation and ownership, such as regular feedback sessions, opportunities for trainees to lead projects, and recognition for outstanding contributions to the program. The COVID-19 pandemic has presented significant challenges to healthcare systems worldwide, including resource constraints, workforce shortages, and the need to adapt rapidly to changing circumstances. The NCC-QIP has had to navigate these challenges while maintaining its commitment to quality improvement and observed a 50% reduction in self-reported QI concerns during the pandemic's peak. It was through reminders and reinforcing our mission, vision, and values that we were able to get back on track with our QI mission.

Evolution of the Neurocritical Care Quality Program

Continuous evolution and iterative growth are essential to the success and development of any program. The NCC-QIP has undergone significant changes and enhancements over the past 12 years, adapting to the evolving needs of patients and healthcare providers. Some vital programmatic improvements to the data repository include migrating the QI data from a Microsoft Excel spreadsheet to a more robust and secure REDCap data management system [24]. This transition allowed for improved data integrity, security, and ease of access for the NCC-QIP team. To ensure that the NCC-QIP remains responsive to the needs of patients and healthcare providers, the program began incorporating ongoing patient census, acuity, and outcomes data into the database. This information allows the program to monitor trends in the patient population, identify emerging issues, and target interventions accordingly. Harnessing the power of modern statistical software, the NCC-QIP adopted tools such as RStudio [22] to analyze data and create visual dashboards. These dashboards provide an intuitive and accessible way for the NCCS team and other stakeholders to review key performance indicators (KPIs), track progress, and identify areas for improvement. These enhancements to the NCC-QIP demonstrate the program's commitment to continuous growth and development. By adopting innovative technologies and practices, the NCC-QIP can effectively monitor and improve the care provided to patients with severe neurological disorders, ultimately enhancing patient outcomes, safety, and overall care quality.

Programmatic support

A prior study surveyed the landscape of NCC-QIP and explored challenges in maintaining a successful NCC-QIP [4]. The study findings emphasize the importance of ongoing support from the department and the hospital administration to foster a culture of continuous improvement, ensuring the availability of necessary resources and facilitating effective communication among multidisciplinary teams. Strong institutional backing also plays a pivotal role in driving sustainable change by enabling the development and implementation of evidence-based interventions, providing opportunities for ongoing education and training, and fostering collaboration among healthcare providers. In summary, departmental and hospital support is essential for the success of an NCC-QIP [36], as it creates an environment conducive to continuous learning, improvement, and the pursuit of excellence in patient care. The success of the NCC-QIP is bolstered by the support of the Department of Anesthesiology and Pain Medicine, which provides resources, guidance, and oversight for the program. Furthermore, the Program for Patient Improvement and Quality Systems Optimization (PPIQSO) team [37] assists the NCC-QIP by offering expertise in quality improvement methodologies, data analysis, and process optimization. This collaborative approach, combining the backing of a dedicated department with the specialized skills of the PPIQSO team, ensures that the NCC-QIP can thrive and continue to drive meaningful improvements in patient care and outcomes.

Future of the Neurocritical Care QI Program 

As the NCC-QIP looks toward the future, it will focus on embracing value-based care, driving innovations, and adopting a patient-centered approach. By aligning with the latest evidence-based practices and tailoring care delivery to individual patient needs, the program aims to ensure the highest level of care while maximizing cost-effectiveness. Emphasis will be placed on continuous refinement and optimization of treatment protocols, drawing on emerging research and advancements in neurocritical care. Moreover, the program will foster a culture of collaboration, encouraging interdisciplinary teamwork and open communication among healthcare providers to ensure seamless care coordination. By embracing a holistic, patient-centered approach that encompasses physical, emotional, and social aspects of care, the NCC-QIP also aims to promote overall patient and provider well-being and satisfaction. Ultimately, the future of the NCC-QIP lies in its ability to adapt and evolve in response to the ever-changing landscape of healthcare while remaining steadfast in its commitment to delivering the highest quality of care for patients with complex neurological conditions.

A vital aspect of the NCC-QIP's future is the engagement of trainees, who are crucial in shaping the next generation of neurocritical care healthcare providers. To foster a culture of continuous learning and improvement, the program will continue to employ innovative strategies to engage trainees, such as interactive educational modules, hands-on workshops, and mentorship opportunities. Creating educational modules tailored to the unique needs of trainees will not only enhance their understanding of neurocritical care principles but also promote a strong foundation in quality improvement methodologies. These modules may include case-based learning, simulations, and web-based platforms that facilitate easy access and foster active participation. Furthermore, the program will prioritize providing basic and advanced training in designing and implementing QI projects. This will empower trainees to identify areas of improvement, develop targeted interventions, and monitor the impact of their initiatives on patient care and outcomes. By offering structured guidance and support, the NCC-QIP can help trainees gain valuable experience and cultivate the essential skills needed to become effective change agents in their clinical practice. Through this comprehensive approach to trainee engagement, the NCC QI program aims to inspire a new generation of healthcare providers who are well-versed in the latest advancements in neurocritical care and committed to driving meaningful improvements in patient care and safety.

Limitations 

The study has some strengths and limitations. The limitations are typical of a single-center study in that data may not be generalizable to neurocritical care units worldwide. The benchmarks used in our study are specific to the measures selected and may not be the same across all intensive care units and in other countries. The self-reported QI concerns may be unique to our structure of the neurocritical care service, including our management model. We did not perform sample size and power analysis for the data presented. The strength of the study is that this is a large sample study that spans a large period of time. This review allowed us to establish a benchmark, and we look forward to improving in areas that are identified during this gap analysis in an interactive manner.

## Conclusions

A dedicated NCC-QIP can be successfully implemented at a quaternary academic medical center. It is possible to monitor and review a large volume of neurocritical care patients. The three most reported NCC-QI concerns may be related to care coordination-communication/handoff, medication-related concerns, and equipment/devices-related complications. The documentation of illness severity scores and stroke measures depends upon the type of measure and ability to reliably and accurately abstract and can be challenging. The quality improvement process can be enhanced by the development and implementation of educational modules that reinforce neurocritical care quality and safety.
